# Dual SMO/BRAF Inhibition by Flavonolignans from *Silybum marianum* †

**DOI:** 10.3390/antiox9050384

**Published:** 2020-05-05

**Authors:** Antonia Diukendjieva, Maya M. Zaharieva, Mattia Mori, Petko Alov, Ivanka Tsakovska, Tania Pencheva, Hristo Najdenski, Vladimír Křen, Chiara Felici, Francesca Bufalieri, Lucia Di Marcotullio, Bruno Botta, Maurizio Botta, Ilza Pajeva

**Affiliations:** 1 Department of QSAR and Molecular Modelling, Institute of Biophysics and Biomedical Engineering, Bulgarian Academy of Sciences, 1113 Sofia, Bulgaria; antonia.diukendjieva@biomed.bas.bg (A.D.); petko.alov@biophys.bas.bg (P.A.); itsakovska@biomed.bas.bg (I.T.); tania.pencheva@biomed.bas.bg (T.P.); 2 Department of Infectious Microbiology, Stephan Angeloff Institute of Microbiology, Bulgarian Academy of Sciences, 1113 Sofia, Bulgaria; zaharieva26@gmail.com (M.M.Z.); hnajdenski@abv.bg (H.N.); 3 Department of Biotechnology, Chemistry and Pharmacy, Department of Excellence 2018-2022, University of Siena, 53100 Siena, Italy; maurizio.botta@unisi.it; 4 Laboratory of Biotransformation, Institute of Microbiology of the Czech Academy of Sciences, 14220 Prague, Czech Republic; kren@biomed.cas.cz; 5 Department of Molecular Medicine, Sapienza University of Rome, 00161 Rome, Italy; chiarafelici5@gmail.com (C.F.); francesca.bufalieri@uniroma1.it (F.B.); 6 Department of Molecular Medicine, Laboratory Affiliated to Istituto Pasteur Italia Fondazione Cenci Bolognetti, Sapienza University of Rome, 00161 Rome, Italy; lucia.dimarcotullio@uniroma1.it; 7 Department of Chemistry and Technology of Drugs, Sapienza University of Rome, 00185 Rome, Italy; bruno.botta@uniroma1.it

**Keywords:** silybins, BRAF kinase, Smoothened, in silico methods, cytotoxicity

## Abstract

Silymarin is the standardized extract from the fruits of *Silybum marianum* (L.) Gaertn., a well-known hepatoprotectant and antioxidant. Recently, bioactive compounds of silymarin, i.e., silybins and their 2,3-dehydro derivatives, have been shown to exert anticancer activities, yet with unclear mechanisms. This study combines in silico and in vitro methods to reveal the potential interactions of optically pure silybins and dehydrosilybins with novel protein targets. The shape and chemical similarity with approved drugs were evaluated in silico, and the potential for interaction with the Hedgehog pathway receptor Smoothened (SMO) and BRAF kinase was confirmed by molecular docking. In vitro studies on SMO and BRAF V600E kinase activity and in BRAF V600E A-375 human melanoma cell lines were further performed to examine their effects on these proteins and cancer cell lines and to corroborate computational predictions. Our in silico results direct to new potential targets of silymarin constituents as dual inhibitors of BRAF and SMO, two major targets in anticancer therapy. The experimental studies confirm that BRAF kinase and SMO may be involved in mechanisms of anticancer activities, demonstrating dose-dependent profiles, with dehydrosilybins showing stronger effects than silybins. The results of this work outline the dual SMO/BRAF effect of flavonolignans from *Silybum marianum* with potential clinical significance. Our approach can be applied to other natural products to reveal their potential targets and mechanism of action.

## 1. Introduction

Historically, natural products (NPs) have been a rich source of various medicinal preparations, and nowadays, they continue to provide leads for compounds that enter clinical trials, mainly as anticancer and antimicrobial agents [1]. Indeed, about one-third of the FDA-approved drugs over the past 20 years are based on NPs or their derivatives [2]. Recently, the intensity of NP-based drug discovery has been reinforced even more by the advent of highly multidisciplinary approaches to evaluating the therapeutic properties of lead compounds and to studying their molecular effects in physiological conditions.

*Silybum marianum* (L.) Gaertn. is a well-known medicinal plant, used since ancient times for the treatment of liver and gallbladder disorders of different etiologies. The active constituent of the herb, i.e., silymarin, is a mixture of polyphenolic compounds such as silybin, isosilybin, dehydrosilybin, silychristin, and silydianin, which are mainly found in its fruit and seeds [3,4]. Silybin, the major flavonolignan component of silymarin, and its 2,3-dehydro derivative dehydrosilybin naturally occur as pairs of stereomers, denoted A and B [4]. Compared to silybin, the amount of 2,3-dehydrosilybin is much lower; however, both, 2,3-dehydrosilybin A and B are present in the silymarin preparations [5] and their content could reach several percent of the total composition depending on the sample origin [6,7].

The initial mechanistic studies of silymarin effects in carbon tetrachloride-induced liver damage attributed its protective action to antioxidant properties [8,9]. Recent studies have indicated a number of new promising effects of silymarin components related to neurological diseases, such as Alzheimer’s [10] and Parkinson’s disease [11], metabolic syndrome [12], and cancer [13]. Silybin improves glycemic homeostasis by positively affecting the activity of pancreatic β-cells, increasing insulin sensitivity of liver and muscle cells, while decreasing lipid deposition in adipocytes [14]. With respect to cancer, silybin has been shown to inhibit various cancer cell types by modulating multiple processes, including growth inhibition, inhibition of angiogenesis, chemosensitization, and modulation of metastatic capacity [15]. Furthermore, a growing body of evidence demonstrates the higher potency of silybin dehydro-derivatives in various experimental settings related to therapeutic usefulness [16].

The broad spectrum of biological activities of silymarin components suggests their potential as lead compounds in the context of multifaceted pathologies such as cancer and metabolic syndrome and offers an attractive possibility to further enhance the therapeutic potential of these molecules through suitable chemical modifications of their structure. Moreover, silymarin has shown favorable safety profiles and is well tolerated at therapeutic doses [17]. In line with this prospect, there is a need for more focused efforts on elucidating the mechanisms of action as well as the relevant targets of flavonolignans in the context of human pathologies.

This study combines in silico and in vitro methods in order to give insights into the possible interactions of flavonolignans from *Silybum marianum* with target proteins endowed with therapeutic implications. The chemical similarity between silybin and 2,3-dehydrosilybin diastereoisomers and approved drugs from the DrugBank database [18] was evaluated, while the potential for the interaction with targets of chemically similar anticancer drugs (Smootened (SMO) and BRAF kinase) was confirmed by molecular docking. Further, we performed in vitro studies of the effects of flavonolignans on mechanisms including the targets of the corresponding drugs—BRAF V600E kinase activity, the viability of A-375 human melanoma cells (with BRAF V600E mutation), and Hedgehog (HH) signaling pathway, including SMO.

## 2. Materials and Methods

### 2.1. Chemicals 

**Figure 1:**
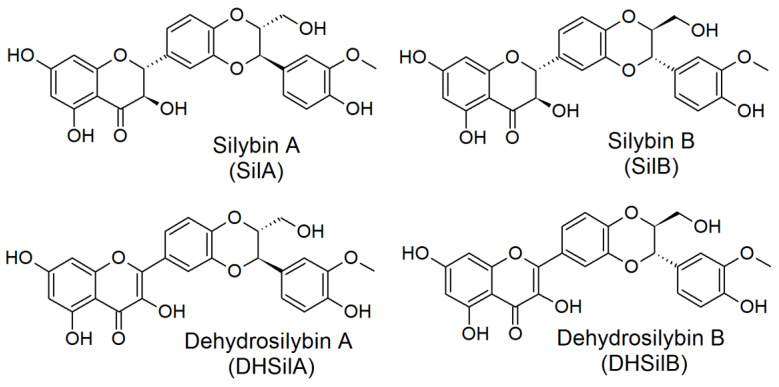
Chemical structures of the studied flavonolignans.

Four compounds (Figure 1), provided by the Laboratory of Biotransformation, Institute of Microbiology of the Czech Academy of Sciences, Prague, were investigated in vitro: silybin A, silybin B [19], 2,3-dehydrosilybin A, and 2,3-dehydrosilybin B [20]. Optically pure diastereoisomers were studied as it had already been shown that stereochemistry was pivotal for the biological activities of flavonolignans [21,22]. The purity of flavonolignans was above 96% (HPLC/PDA).

### 2.2. Similarity Assessment with ROCS

The chemical similarity between silybin and dehydrosilybin stereomers and approved drugs from the DrugBank database [18] was evaluated by the ROCS (Rapid Overlay of Chemical Structures) software (OpenEye 2019.05, Santa Fe, NM, USA) [23]. ROCS is a tool for aligning and scoring a database of molecular conformers to a query or template of molecule conformers by utilizing the idea that molecules’ shape similarity could be inferred from their volumes overlay. These scores may then be used to estimate the probability that molecules share relevant features with a query. For this purpose, 3D conformers of the four flavonolignans and the approved drugs from the DrugBank database were generated using Open Eye OMEGA in ROCS mode with the following relevant parameters: the maximum number of conformations = 50, the root-mean-square distance for duplicates = 0.5 Å, and energy window = 10 kcal/mol. ROCS was run in multiconformer query mode with flavonolignans as query molecules.

### 2.3. Docking

The Molecular Operating Environment (MOE 2019.0102) software (Montreal, Canada) [24] was used for docking studies in the binding pockets of SMO (PDB ID: 5L7I, Structure of human Smoothened in complex with vismodegib, chain A) and BRAF kinase (PDB ID: 4RZV, Crystal structure of the BRAF (R509H) kinase domain monomer bound to vemurafenib, chain A). The X-ray structures were initially prepared using the MOE tool “Protonate3D”. During the preparation, physiologically relevant parameters were set as follows: temperature = 310 K, pH = 7.4, ion concentration = 0.152 mol/L. The docking site was defined by ligands’ atoms. The triangle placement method and the London dG scoring function were applied to score the generated poses of the docked ligands. The best 10 poses per compound were retained and further analyzed using the MOE tool “Ligand Interactions”.

### 2.4. HH-Dependent Luciferase Reporter Assay

The luciferase assay was carried out in NIH3T3 Shh-Light II cells, stably incorporating a Gli-responsive luciferase reporter and the pRL-TK Renilla, treated with SAG (SMO Agonist, 200 nM, Alexis Biochemicals Farmingdale, NY, USA) and the selected compounds for 48 h. Luciferases activities from *P. pyralisand* and *R. reniformis* were assayed with a dual-luciferase assay system, according to the manufacturer’s instructions (Biotium Inc., Hayward, CA, USA). The results were expressed as a *P. pyralisand*/*R. reniformis* luciferase ratio and represent the mean ± S.D. of three experiments, each performed in triplicate.

### 2.5. BODIPY-Cyclopamine Binding Assay

Human Myc-DDK-tagged SMO or empty vector was transfected in HEK293T cells. Cells were washed in phosphate-buffered saline (PBS) supplemented with 0.5% fetal bovine serum, fixed in 4% paraformaldehyde in PBS for 10 min, and incubated for 2 h at 37 °C, both in the same medium supplemented with BODIPY-Cyclopamine (BioVision Inc., San Francisco, CA, USA) (BC, 5 nM) and the studied compounds. The cells were permeabilized with Triton X100 (Sigma-Aldrich, St. Louis, MO, USA) 0.2%, and Hoechst reagent was used for cell nuclei staining. Data were expressed as a percentage of BC incorporation observed with BC alone [25].

### 2.6. BRAF Kinase Assay

The activities of silybins and dehydrosilybins were tested using a commercial BRAF (V600E) (serine/threonine-protein kinase B-raf, V600E mutant) Kinase Assay Kit (#48688, BPS Bioscience, San Diego, CA, USA). Briefly, the BRAF (V600E) enzyme was incubated for 45 min at 30 °C with five different concentrations of each tested compound in logarithmic dilutions (1000, 100, 10, 1, and 0.1 µM). The reaction mixture was prepared following the instructions of the producer. The luminescence of the product of the enzyme activity was measured with the Kinase-Glo MAX Kit (#V6071, Promega, Madison, WI, USA) at a microplate luminometer (Synergy Multi-Mode Reader, BioTek, Winooski, VT, USA). Higher luminescent signal corresponded to higher enzyme activity. Vemurafenib (PLX4032, #S1267, Selleck Chemicals, Houston, TX, USA) applied in concentrations 0.1, 1, 10, 100, and 1000 nM served as a reference compound. The reaction mixture without the test inhibitor represented the positive control, whereas the mixture without the test inhibitor and without the enzyme was used as blank. 

### 2.7. Cytotoxicity Assay

The cytotoxic activity of the tested compounds was evaluated on non-tumorigenic human keratinocytes (HaCaT, #300493), skin melanoma malignant cells with BRAF V600E mutation (A-375, #300110), and skin epidermoid carcinoma cells (A-431, #300112), all purchased from CLS Cell Lines Service (Eppelheim, Germany). The cell lines were maintained in DMEM (#DMEM-HPA, Capricorn^®^, Ebsdorfergrund, Germany) supplemented with 10% fetal bovine serum (#FBS-HI-12A, Capricorn^®^, Ebsdorfergrund, Germany) and 4 mM L-glutamine (#G7513, Merck (Sigma-Aldrich), Germany) and incubated under standard conditions (5% CO_2_, 37 °C, 90%–95% humidity). The experiments were performed between passages 4 and 15 whereby the cells were sub-cultured every 4th day by splitting 1:8, according to the protocol of the biobank. Briefly, the cell cultures were rinsed with PBS without calcium and magnesium (PBS, #D8537, Merck (Sigma-Aldrich), Germany) and incubated for 8–10 min with accutase (#ACC-1B, Capricorn^®^, Ebsdorfergrund, Germany) as a detachment agent. After centrifugation, the cells were counted with trypan blue (0.4% solution, #T8154, Merck (Sigma-Aldrich), Germany).

The antiproliferative effects of SilA, SilB, DHSilA, and DHSilB were tested using the MTT-dye (3-(4,5-dimethylthiazol-2-yl)-2,5-diphenyltetrazolium bromide) assay according to ISO 10993-5-2009, Annex C [26]. Briefly, the cells were seeded in 96-well plates at a density of 0.1 × 10^6^/mL, incubated for 24 h at 37 °C (5% CO_2_), and treated with ten concentrations of the compounds ranging from 3.9 to 1000 µM in twofold serial dilutions. After 72 h, the MTT-dye (#M5655-1G, Merck (Sigma-Aldrich), Germany) was added to each sample at a final concentration of 0.05 mg/mL and the plates were incubated for 2 h at 37 °C (5% CO_2_). The supernatant was aspirated, and the formazan crystals formed as a product of the enzymatic MTT reduction were dissolved in 2-propranol (#33539, Merck (Sigma-Aldrich), Germany) supplemented with 5% formic acid (#300415, ChimSpectar/Neuber, Sofia, Bulgaria). The absorbance of the product was measured at 550 nm (reference filter 690 nm) on an ELISA Reader BioTek *ELx*800 (BioTek).

### 2.8. IC_50_ Values and Statistical Analysis

The experiments for evaluation of the BRAF inhibition and cytotoxicity were performed in triplicate. The IC_50_ values were calculated by the BfROpenLab’s non-linear regression nodes in the KNIME analytics platform [27] with the constant slope model.

## 3. Results and Discussions

### 3.1. In Silico Studies

The chemical similarity between silybin and dehydrosilybin stereoisomers and approved drugs from the DrugBank database was assessed using the ROCS software (OpenEye) [28]. The TanimotoCombo index (TCI) was used as a similarity metrics as it combines the shape and chemical features based on the alignment between known drugs and silybin and dehydrosilybin queries. Given the known anticancer effect of flavonolignans such as silybins, the structural and chemical similarity of these molecules with respect to drugs exerting clinical antitumor activity becomes of particular interest to predict the mechanism of action of these compounds. Therefore, we filtered and analyzed anticancer drugs for which similarity with silybin and dehydrosilybin isomers was scored with TCI ≥ 0.9 (Table 1). Vemurafenib is approved for the treatment of metastatic melanoma as a competitive inhibitor of BRAF kinase bearing a substitution of glutamic acid for valine (V600E mutation) [29]. Vismodegib selectively binds to and inhibits the transmembrane receptor SMO, i.e., the upstream regulator of the HH signaling pathway, and it is indicated by the FDA for the treatment of metastatic or locally advanced basal cell carcinoma [30]. ROCS results showed slightly higher TCI values for dehydrosilybins compared to silybins with respect to both vemurafenib and vismodegib.

Based on the observed similarity, we can assume that the studied flavonolignans may interact with the same targets that these antitumor drugs do. To examine this hypothesis, the potential interactions with targets of vemurafenib and vismodegib, SMO, and BRAF kinase, respectively, were further studied with in silico methods. Specifically, molecular docking simulations were carried out using high-resolution X-ray structures of these proteins available in the Protein Data Bank.

**Figure 2:**
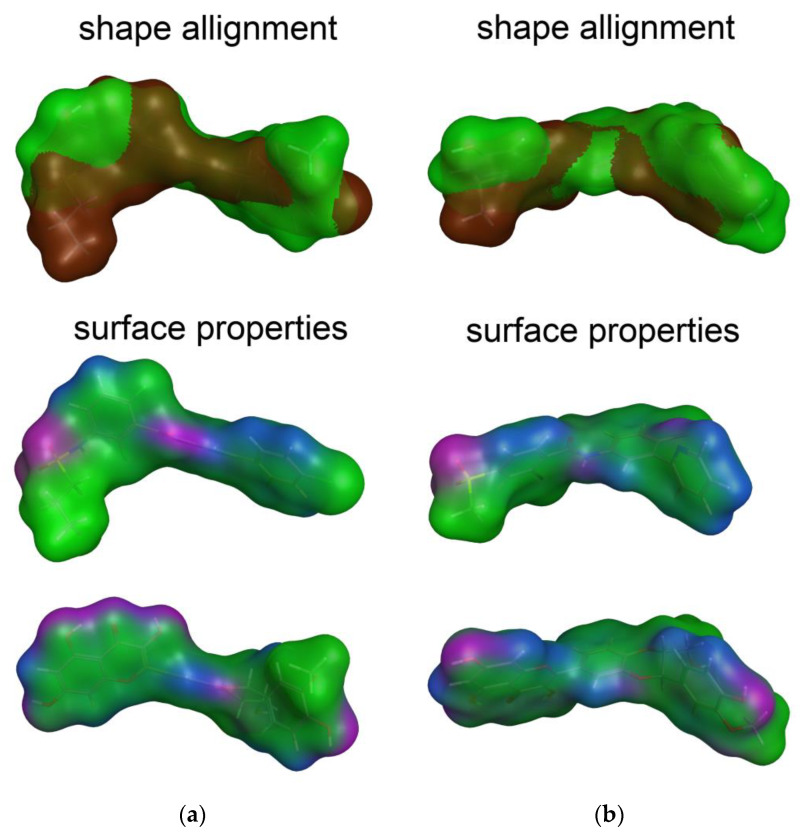
The best scored alignments of: (**a**) DHSilB and vemurafenib; (**b**) DHSilA and vismodegib. Shape alignment (color legend): vemurafenib and vismodegib—brown, DHSilA and DHSilB—green. Surface properties (color legend): H-bonding (magenta), mild polar (blue), and hydrophobic (green).

Figure 2 illustrates the similarity in the shape and molecular surface properties separately for DHSilB and vemurafenib (a), and DHSilA and vismodegib (b). 

The docking results demonstrate that silybins and dehydrosilybins can be accommodated into the binding sites of the studied targets. Docking poses were further analyzed in relation to the best correspondence of flavonolignans to the X-ray ligands in the protein pocket, and their interaction energies with the receptor. The results are summarized in Table 2. The most reasonable docking poses of dehydrosilybins yielded better docking scores than silybins in both receptors. For BRAF kinase, the docking score of DHSilB is the lowest (−8.354), compared to the other flavonolignans, although it is higher than the redocked score of vemurafenib. Regarding SMO, DHSilA and DHSilB have the lowest docking scores, comparable to that of vismodegib.

**Figure 3:**
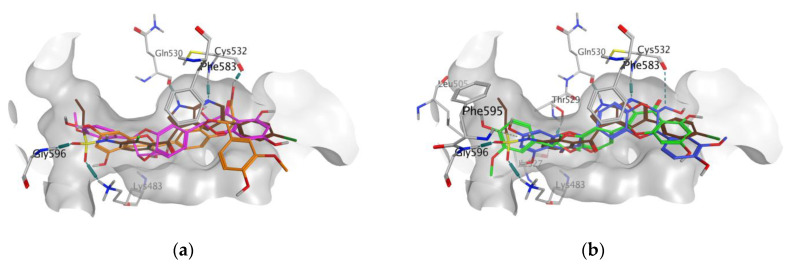
Poses and interactions of the studied compounds in the BRAF kinase binding pocket: (**a**) SilA (magenta) and SilB (orange); (**b**) DHSilA (blue) and DHSilB (green). In both panels, vemurafenib is shown in brown, the pocket is shown in grey, and the interacting protein residues are colored in atom types.

**Figure 4:**
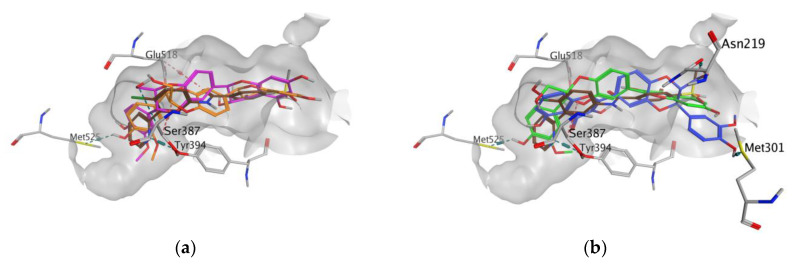
Poses and interactions of the studied compounds in the SMO binding pocket: (**a**) SilA (magenta) and SilB (orange); (**b**) DHSilA (blue) and DHSilB (green). In both panels, vismodegib is shown in brown, the pocket is shown in grey, and the interacting protein residues are colored in atom types.

In all docking simulations, we identified the binding site residues involved in specific interactions with flavonolignans, including those involved in interactions with vemurafenib (Lys483, Cys532) and vismodegib (Ser387) (Figure 3 and Figure 4). We also observed multiple interactions that differed from those specified for the approved anticancer drugs, which were particularly evident for dehydrosilybins in both receptors. Specifically, DHSilA performs an aromatic interaction with Phe583, while DHSilB interacts through a hydrogen bond with Thr529 and Ile527, as well as through an aromatic interaction with Phe595 in the BRAF kinase. Notably, despite the comparable docking scores, only DHSilA forms two hydrogen bonds with the residues Asn219 and Met301 in the binding site of SMO (Figure 4a). Inverted (mirror-like) poses were observed for all diastereoisomer pairs in both proteins, with the exception of SilA and SilB in SMO. Such stereospecific orientation of the compounds in the binding sites of the studied proteins is not surprising, considering the ability of these compounds to interact in a stereospecific manner with other receptors [22]. This also clearly demonstrates the utmost importance of working with the pure stereomers of these flavonolignans.

### 3.2. In Vitro Studies

The results of the in silico study pointed to the flavonolignans’ potential to interact with the identified anticancer drug targets. Therefore, in vitro experiments were further performed to examine their possible effects on these proteins and cancer cell lines, and to possibly corroborate computational predictions. With respect to BRAF kinase, we studied the effects of silybins on BRAF V600E kinase activity as well as on the viability of human malignant melanoma cells expressing BRAF V600E kinase and non-tumorigenic skin cells. As for SMO, the effects on the HH pathway were investigated together with the potential interaction with the SMO receptor. Since it is anticipated that flavonolignans may act as inhibitors of the target proteins, like chemically similar drugs do, assays for testing inhibitory activity were selected for this purpose.

**Figure 5:**
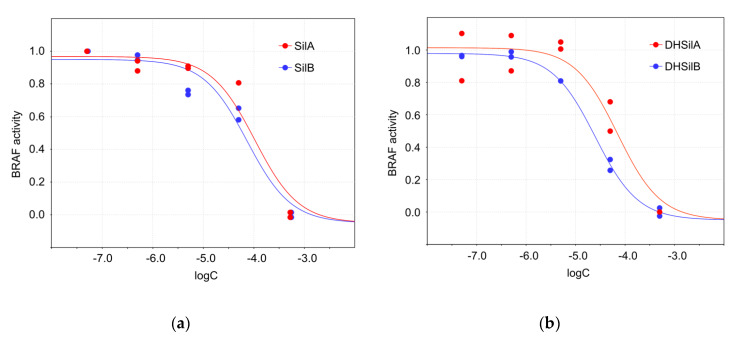
Inhibitory effects of the studied compounds on BRAF V600E kinase activity: (**a**) SilA (IC_50_ = 104.0 µM, 95%, confidence interval, CI = 34.7 ÷ 204.2 µM) and SilB (IC_50_=73.9 µM, CI = 32.4 ÷ 112.2 µM); (**b**) DHSilA (IC_50_ = 70.6 µM, CI = 30.9 ÷ 131.8 µM) and DHSilB (IC_50_ = 24.9 µM, CI = 17.8 ÷ 26.3 µM).

Studies of the effects of silybins on the activity of BRAF V600E kinase activity demonstrate that DHSilB exhibits the highest inhibitory activity, with an IC_50_ of 24.9 µM, followed by DHSilA, SilB, and SilA (Figure 5). As for comparison, the reported IC_50_ value of vemurafenib is 32.4 nM.

Regarding cell viability, all tested flavonolignans exhibited higher cytotoxicity in the A-375 cell line, which is representative of malignant melanoma expressing the BRAF V600E mutation, compared to the non-melanoma tumor cell line A-431 and the non-tumorigenic cell line HaCaT. The IC_50_ values obtained for the three tested cell lines are reported in Table 3. DHSilA and DHSilB exhibit higher cytotoxicity toward the A-375 cell line and appear to be less toxic in A-431 and HaCaT cell lines compared to SilA and SilB. This trend is especially pronounced for the cell line HaCaT, in which dehydrosilybins exhibit the lowest toxicity.

**Figure 6:**
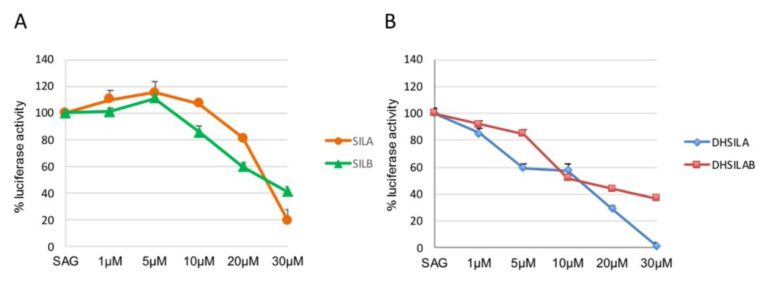
Hedgehog signaling pathway inhibitory activity of: (**A**) SilA and SilB(A); (**B**) DHSilA and DHSilAB.

With respect to the SMO receptor, the HH inhibitory activity of the compounds, alone and in a racemate, was investigated in a luciferase reporter assay, which is widely used for characterizing HH inhibitors. The HH inhibitory activity of synthesized molecules was evaluated in NIH3T3 Shh Light II cells, stably incorporating a Gli-responsive firefly luciferase [31] treated with the synthetic SMO agonist SAG [32], alone and in combination with the tested compounds. At the maximum concentration of 30 μM, SilA and SilB (Figure 6A) showed mild activity as HH inhibitors, while DHSilA and the racemate of DHSilA and DHSilB (namely, DHSilAB) showed high activity in this assay, having an IC_50_ of 5–10 μM (Figure 6B). The remaining SilAB and DHSilB proved to be inactive.

**Figure 7:**
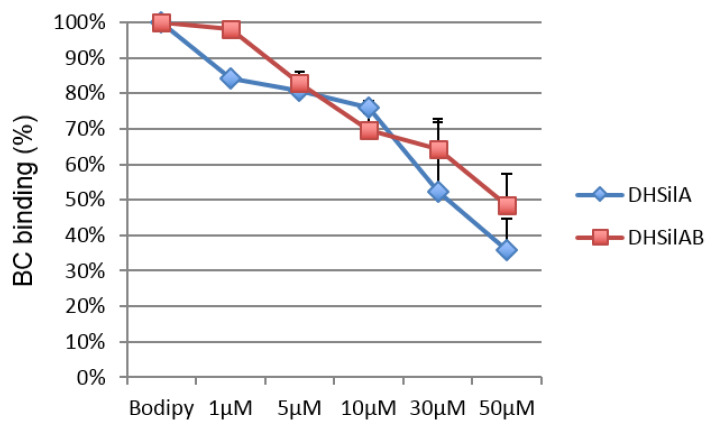
Inhibition of BODIPY-Cyclopamine binding by DHSilA and DHSilB.

To further investigate the binding properties of the selected compounds to the SMO receptor, we performed a displacement assay based on the use of BODIPY-Cyclopamine [33,34], which is known to interact with the binding site of SMO antagonists located within the heptahelical bundle of the receptor. To this aim, HEK293T cells were transiently transfected for expression of SMO and then incubated with BC in the presence or absence of increased amounts of DHSilA or DHSilAB. These compounds inhibited BC binding to cells expressing SMO in a dose-dependent manner (Figure 7).

Overall, our findings have shown that DHSilA and DHSilAB bind the SMO receptor at the level of its heptahelical bundle, which is supportive of the SMO antagonism effect, as previously observed for other natural product chemotypes [35,36].

## 4. Conclusions

The combined in silico and in vitro approach applied in this study points to novel anticancer targets for flavonolignans from *Silybum marianum* (L.) Gaertn. The in silico simulations helped in the identification of novel potential protein targets relying on: (i) the chemical similarity of the studied compounds to the well-known antitumor drugs vemurafenib and vismodegib, and (ii) the ability of flavonolignans to interact with the targets of these drugs, proved by molecular docking simulations. The in silico results suggest that the active components of silymarin may act as dual inhibitors of BRAF kinase and SMO, two major targets in current anticancer therapy. 

In vitro assays were further performed on the proteins and cell lines, outlining dose-dependent profiles of the studied compounds and suggesting their possible effects on these targets. As a whole, we observed a good consistency between the results from the in silico simulations and the in vitro experiments. The docking scores as a measure of the ligand-receptor interaction energy illustrate a good correspondence with IC_50_ values determined in the in vitro assays (Table 2, Table 3, and Figure 5). Clearly, dehydrosilybins yield better docking values compared to silybins, and they are the more active compounds in the BRAF kinase and HH pathway assays. This observation is in agreement with other in vitro studies confirming the higher activity of dehydrosilybins in comparison with silybins [16]. The cytotoxicity experiments additionally corroborate the cytotoxic properties of the compounds in malignant skin cell lines demonstrating higher activity in A-375 cells than in A-431 and HaCaT.

In summary, our study confirms that BRAF kinase and SMO receptor may be involved in the mechanisms of the anticancer activities of flavonolignans from *Silybum marianum* (L.) Gaertn and outlines dehydrosilybins as potential lead structures for the development of anticancer drugs. Furthermore, it demonstrates the efficacy of the applied combined in silico/in vitro approach to facilitate the identification of the potential targets of bioactive compounds from natural sources and to help in elucidating their molecular mechanism of action. The results of this study pave the way to further structure-guided optimization of flavolignans from *Silyum marianum* as anticancer leads. In addition, they may be a profitable case study in the identification of the possible mechanism of action of other bioactive compounds.

## Figures and Tables

**Table 1:** TanimotoCombo indices for antitumor drugs whose similarity with silybin and dehydrosilybin stereomers is scored higher than 0.9.

	**Vemurafenib**	**Vismodegib**
	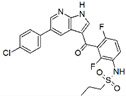	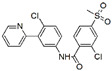
	**TanimotoCombo Index**	
SilA	0.963	0.962
SilB	0.943	0.962
DHSilA	0.976	0.979
DHSilB	0.963	0.979

**Table 2:** Docking scores (kcal/mol) of silybins, dehydrosilybins, vemurafenib, and vismodegib (docking in BRAF kinase and SMO; the lower scores suggest higher binding affinities).

Compound	BRAF Kinase	Smoothened Receptor
SilA	−5.787	−7.928
SilB	−6.158	−5.545
DHSilA	−7.696	−8.090
DHSilB	−8.354	−8.490
Vemurafenib	−10.196	N.A.
Vismodegib	N.A.	−8.429

**Table 3:** In vitro cytotoxicity of silybins and dehydrosilybins on malignant skin cell lines and non-tumorigenic keratinocytes.

Compound	IC_50_ [µM] (95% Confidence Interval)		
	Cell Lines		
	A-375	A-431	HaCaT
SilA	97.0 (38.0 ÷ 245.5)	126.0 (51.3 ÷ 288.4)	120.0 (56.2 ÷ 245.5)
SilB	120.0 (53.7 ÷ 257.0)	166.0 (52.5 ÷ n.d.)	150.0 (57.5 ÷ 426.6)
DHSilA	83.0 (44.7 ÷ 158.5)	97.0 (55.0 ÷ 169.8)	231.0 (91.2 ÷ 457.1)
DHSilB	86.0 (64.6 ÷ 120.2)	130.0 (79.4 ÷ 213.8)	164.0 (75.9 ÷ 309.0)

## Abbreviations

DHSilA	dehydrosilybin A
DHSilB	dehydrosilybin B
HH	Hedgehog signalling pathway
MTT	3-(4,5-dimethylthiazol-2-yl)-2,5-diphenyltetrazolium bromide
NP	natural products
SilA	silybin A
SilB	silybin B
SAG	Smoothened Agonist
SMO	Smoothened
TCI	TanimotoCombo index

## References

[1] Harvey A.L., Edrada-Ebel R., Quinn R.J. (2015). The re-emergence of natural products for drug discovery in the genomics era. Nat. Rev. Drug Discov..

[2] Patridge E., Gareiss P., Kinch M.S., Hoyer D. (2016). An analysis of FDA-approved drugs: Natural products and their derivatives. Drug Discov. Today.

[3] Chambers C.S., Holečková V., Petrásková L., Biedermann D., Valentová K., Buchta M., Křen V. (2017). The silymarin composition…and why does it matter?. Food Res. Int..

[4] Pyszková M., Biler M., Biedermann D., Valentová K., Kuzma M., Vrba J., Ulrichová J., Sokolová R., Mojović M., Popović-Bijelić A. (2016). Flavonolignan 2,3-dehydroderivatives: Preparation, antiradical and cytoprotective activity. Free Radic. Biol. Med..

[5] Gazak R., Svobodova A., Psotová J., Sedmera P., Přikrylová V., Walterová D., Křen V. (2004). Oxidised derivatives of silybin and their antiradical and antioxidant activity. Bioorg. Med. Chem..

[6] Fenclová M., Novaková A., Viktorová J., Jonatová P., Džuman Z., Ruml T., Křen V., Hajšlová J., Vítek L., Stranská-Zachariášová M. (2019). Poor chemical and microbiological quality of the commercial milk thistle-based dietary supplements may account for their reported unsatisfactory and non-reproducible clinical outcomes. Sci. Rep..

[7] Petrásková L., Káňová K., Valentová K., Biedermann D., Křen V. (2020). A Simple and rapid HPLC separation and quantification of flavonoid, flavonolignans, and 2,3-dehydroflavonolignans in silymarin. Foods.

[8] Rauen H.M., Schriewer H., Tegtbauer U., Lasana J.F. (1973). Silymarin verhindert die Lipidperoxidation bei der CCl_4_-Leberschädigung [Silymarin prevents lipid peroxidation in CCl_4_ liver damage]. Experientia.

[9] Bindoli A., Cavallini L., Siliprandi N. (1977). Inhibitory action of silymarin of lipid peroxide formation in rat liver mitochondria and microsomes. Biochem. Pharmacol..

[10] Yaghmaei P., Azarfar K., Dezfulian M., Ebrahim-Habibi A. (2014). Silymarin effect on amyloid-β plaque accumulation and gene expression of APP in an Alzheimer’s disease rat model. DARU J. Pharm. Sci..

[11] Ullah H., Khan H. (2018). Anti-Parkinson potential of silymarin: Mechanistic insight and therapeutic standing. Front. Pharmacol..

[12] Tajmohammadi A., Razavi B.M., Hosseinzadeh H. (2018). *Silybum marianum* (milk thistle) and its main constituent, silymarin, as a potential therapeutic plant in metabolic syndrome: A review: *Silybum marianum* and Metabolic Syndrome. Phytother. Res..

[13] Polachi N., Bai G., Li T., Chu Y., Wang X., Li S., Gu N., Wu J., Li W., Zhang Y. (2016). Modulatory effects of silibinin in various cell signaling pathways against liver disorders and cancer—A comprehensive review. Eur. J. Med. Chem..

[14] Chu C., Li D., Zhang S., Ikejima T., Jia Y., Wang D., Xu F. (2018). Role of silibinin in the management of diabetes mellitus and its complications. Arch. Pharm. Res..

[15] Millimouno F.M., Dong J., Yang L., Li J., Li X. (2014). Targeting apoptosis pathways in cancer and perspectives with natural compounds from mother Nature. Cancer Prev. Res..

[16] Agarwal C., Wadhwa R., Deep G., Biedermann D., Gažák R., Křen V., Agarwal R. (2013). Anti-cancer efficacy of silybin derivatives—A structure-activity relationship. PLoS ONE.

[17] Soleimani V., Delghandi P.S., Moallem S.A., Karimi G. (2019). Safety and toxicity of silymarin, the major constituent of milk thistle extract: An updated review. Phytother. Res..

[18] Wishart D.S., Knox C., Guo A.C., Shrivastava S., Hassanali M., Stothard P., Chang Z., Woolsey J. (2006). DrugBank: A comprehensive resource for in silico drug discovery and exploration. Nucleic Acids Res..

[19] Biedermann D., Vavříková E., Cvak L., Křen V. (2014). Chemistry of silybin. Nat. Prod. Rep..

[20] Gažák R., Trouillas P., Biedermann D., Fuksová K., Marhol P., Kuzma M., Křen V. (2013). Base-catalyzed oxidation of silybin and isosilybin into 2,3-dehydro derivatives. Tetrahedron Lett..

[21] Gažák R., Walterová D., Křen V. (2007). Silybin and silymarin—new and emerging applications in medicine. Curr. Med. Chem..

[22] Diukendjieva A., Al Sharif M., Alov P., Pencheva T., Tsakovska I., Pajeva I. (2017). ADME/Tox properties and biochemical interactions of silybin congeners: in silico study. Nat. Prod. Commun..

[23] Open Eye Scientific Software 2019.05. www.eyesopen.com.

[24] Molecular Operating Environment Software 2019.0102, Chemical Computing Group Inc. www.chemcomp.com.

[25] Manetti F., Faure H., Roudaut H., Gorojankina T., Traiffort E., Schoenfelder A., Mann A., Solinas A., Taddei M., Ruat M. (2010). Virtual screening-based discovery and mechanistic characterization of the acylthiourea MRT-10 family as Smoothened antagonists. Mol. Pharmacol..

[26] International Organization for Standardization (2009). ISO 10993-5 Biological Evaluation of Medical Devices. Part 5: Tests for In Vitro Cytotoxicity.

[27] Berthold M.R., Cebron N., Dill F., Gabriel T.R., Kötter T., Meinl T., Ohl P., Sieb C., Thiel K., Wiswedel B., Preisach C., Burkhardt H., Schmidt-Thieme L., Decker R. (2008). KNIME: The Konstanz Information Miner. Data Analysis, Machine Learning and Applications.

[28] Hawkins P.C.D., Skillman A.G., Nicholls A. (2007). Comparison of shape-matching and docking as virtual screening tools. J. Med. Chem..

[29] Luke J.J., Hodi F.S. (2012). Vemurafenib and BRAF inhibition: A new class of treatment for metastatic melanoma. Clin. Cancer Res..

[30] Sandhiya S., Melvin G., Kumar S.S., Dkhar S.A. (2013). The dawn of hedgehog inhibitors: Vismodegib. J. Pharmacol. Pharmacother..

[31] Taipale J., Chen J.K., Cooper M.K., Wang B., Mann R.K., Milenkovic L., Scott M.P., Beachy P.A. (2000). Effects of oncogenic mutations in Smoothened and Patched can be reversed by cyclopamine. Nature.

[32] Chen J.K. (2002). Inhibition of Hedgehog signaling by direct binding of cyclopamine to Smoothened. Genes Dev..

[33] Alfonsi R., Botta B., Cacchi S., Di Marcotullio L., Fabrizi G., Faedda R., Goggiamani A., Iazzetti A., Mori M. (2017). Design, Palladium-catalyzed synthesis, and biological investigation of 2-substituted 3-aroylquinolin-4(1*H*)-ones as inhibitors of the Hedgehog signaling pathway. J. Med. Chem..

[34] Infante P., Alfonsi R., Ingallina C., Quaglio D., Ghirga F., D’Acquarica I., Bernardi F., Di Magno L., Canettieri G., Screpanti I. (2016). Inhibition of Hedgehog-dependent tumors and cancer stem cells by a newly identified naturally occurring chemotype. Cell Death Dis..

[35] Berardozzi S., Bernardi F., Infante P., Ingallina C., Toscano S., De Paolis E., Alfonsi R., Caimano M., Botta B., Mori M. (2018). Synergistic inhibition of the Hedgehog pathway by newly designed Smo and Gli antagonists bearing the isoflavone scaffold. Eur. J. Med. Chem..

[36] Lospinoso Severini L., Quaglio D., Basili I., Ghirga F., Bufalieri F., Caimano M., Balducci S., Moretti M., Romeo I., Loricchio E. (2019). A Smo/Gli Multitarget Hedgehog pathway inhibitor impairs tumor growth. Cancers.

